# Lung function tracking in children with perinatally acquired HIV following early antiretroviral therapy initiation

**DOI:** 10.1136/thorax-2023-220197

**Published:** 2023-07-21

**Authors:** André Gie, Claire Davies, Florin Vaida, Julie Morrison, David Maree, Kennedy Otwombe, Sara H Browne, Marieke M van der Zalm, Mark F Cotton, Steve Innes, Pierre Goussard

**Affiliations:** 1 Department of Paediatrics and Child Health, Faculty of Medicine and Health Sciences, Stellenbosch University, Cape Town, South Africa; 2 Division of Epidemiology and Biostatistics, Stellenbosch University, Stellenbosch, South Africa; 3 Division of Biostatistics and Bioinformatics, School of Public Health, University of California, La Jolla, California, USA; 4 Netcare Christiaan Barnard Memorial Hospital, Cape Town, South Africa; 5 Department of Medicine, Stellenbosch University, Stellenbosch, South Africa; 6 School of Public Health, Faculty of Health Sciences University of the Witwatersrand, Johannesburg, South Africa; 7 Perinatal HIV Research Unit, Faculty of Health Sciences University of the Witwatersrand, Johannesburg, South Africa; 8 School of Public Health, University of California, La Jolla, California, USA; 9 Department of Paediatrics and Child Health, Tygerberg Children’s Hospital and Stellenbosch University, Tygerberg, South Africa; 10 Department of Paediatrics and Child Health, Stellenbosch University, Stellenbosch, South Africa; 11 Family Center for Research with Ubuntu, Stellenbosch University, Stellenbosch, South Africa; 12 Desmond Tutu HIV Centre, University of Cape Town, Rondebosch, South Africa

**Keywords:** Paediatric Lung Disaese, Lung Physiology, Respiratory Measurement, Systemic disease and lungs

## Abstract

**Introduction:**

Lung disease remains a frequent complication in children with perinatal HIV infection (CHIV) and exposure without infection (CHEU), resulting in diminished lung function. In CHIV, early antiretroviral therapy (ART) initiation improves survival and extrapulmonary outcomes. However, it is unknown if there is benefit to lung function.

**Methods:**

Cohorts of CHIV (ART initiated at median 4.0 months), CHEU and HIV-unexposed children (CHU) prospectively performed pulmonary function testing (PFT) consisting of spirometry, plethysmography and diffusing capacity from 2013 to 2020. We determined lung function trajectories for PFT outcomes comparing CHIV to CHU and CHEU to CHU, using linear mixed effects models with multiple imputation. Potential confounders included sex, age, height, weight, body mass index z-score, urine cotinine and Tanner stage.

**Results:**

328 participants (122 CHIV, 126 CHEU, 80 CHU) performed PFT (ages 6.6–15.6 years). Spirometry (forced expiratory volume in 1 s, FEV1, forced vital capacity (FVC), FEV1/FVC) outcomes were similar between groups. In plethysmography, the mean residual volume (RV) z-score was 17% greater in CHIV than CHU (95% CI 1% to 33%, p=0.042). There was no difference in total lung capacity (TLC) or RV/TLC z-scores between groups. Diffusing capacity for carbon monoxide was similar in all groups, while alveolar volume (VA) differed between HIV groups by sex.

**Conclusion:**

Our study indicates that early ART initiation can mitigate the loss of lung function in CHIV with lasting benefit through childhood; however, there remains concern of small airway disease. CHEU does not appear to disrupt childhood lung function trajectory.

**WHAT IS ALREADY KNOWN ON THIS TOPIC**

- Lung function abnormalities are frequently found in children with perinatally acquired HIV (CHIV) or exposure without infection (CHEU). Antiretroviral therapy (ART) and vertical transmission prophylaxis have decreased HIV transmission and pulmonary complications including opportunistic infections and bronchiectasis; however, lung function abnormalities remain common and the effect of early ART on lung function is unknown.

**WHAT THIS STUDY ADDS**

- Early ART initiation provides robust lung function benefit that extends throughout childhood and mitigates the diminished lung function associated with perinatal HIV infection. Spirometry is similar throughout childhood in CHIV with early ART to CHEU and children unexposed to HIV. However, plethysmography establishes increased air-trapping in CHIV suggestive of small airway disease. Lung function trajectory is not disrupted by perinatal HIV exposure without infection. Interpretation of lung function is hampered by the lack of reference data for diverse populations, including those of South African and African ancestries, as available reference data may not reflect lung function trajectories in all populations.

**HOW THIS STUDY MIGHT AFFECT RESEARCH, PRACTICE OR POLICY**

- ART should be initiated early in life following perinatal HIV infection to attenuate the loss of lung function related to HIV-associated lung disease.

## Introduction

HIV-associated lung disease has evolved with the introduction of vertical transmission prophylaxis and antiretroviral therapy (ART). Many HIV-exposed children are now uninfected (CHEU), and children with perinatal HIV infection (CHIV) have access to early ART. Nonetheless, lung disease remains a frequent complication following perinatal HIV exposure and infection. Pulmonary infection and associated postinfectious structural consequences, including bronchiectasis, are now less common, however, lung function abnormalities remain prevalent in children perinatally exposed to and infected with HIV.^1–3^

The precise pathophysiology of HIV-associated lung disease and related lung function abnormalities in the presence of ART availability is not fully understood. Antenatal factors including exposure to HIV, ART and an abnormal inflammatory profile may influence lung development in utero in children exposed to HIV.^2 4^ During the first years of life, small airway disease is a frequent abnormality in CHIV resulting in airflow limitation, air-trapping, ventilation inhomogeneity and increased airway resistance.^1 3 5 6^ Small airway disease may be due to reversable small airway obstruction, as found in asthma and bronchial hyperresponsiveness, or due to small airway injury resulting from HIV itself, respiratory infection or dysregulated lung and airway immunity.

While early HIV diagnosis and ART initiation decreases mortality, improves neurocognitive outcome and reduces the HIV reservoir, the influence on childhood lung function is unclear.^7 8^ Available data are limited to cross-sectional studies which demonstrate lung function advantage with early ART.^6 9^ Longitudinal lung function studies are scarce and do not represent the current practice of early HIV diagnosis and ART. Lung function in CHIV that did not benefit from early ART initiation follow a similar, although lower trajectory, to those unexposed to HIV.^10 11^ This suggests that early intervention is required to prevent lung disease.

Increasing evidence demonstrates that childhood lung function and lung function trajectory is determined early in life. Those with poor childhood lung function follow a low trajectory and fail to reach their full lung function potential.^12^ The failure to reach peak adult lung function is a significant risk factor to develop chronic obstructive pulmonary disease (COPD) and cardiorespiratory events later in life.^13 14^ A substantial concern of HIV-associated lung disease in the era of ART availability is that the large population of CHIV will prematurely develop COPD as they age unless therapeutic strategies are identified to improve lung function.

In view of the limited knowledge of the influence of early ART on childhood lung function, we tested the hypothesis that in CHIV, early ART initiation would limit HIV-associated lung disease and preserve lung function, thereby allowing normal lung function development. Our study compares the longitudinal lung function through childhood of CHIV to CHEU and HIV-unexposed children (CHU). Furthermore, to establish the influence of perinatal HIV exposure, we compare longitudinal lung function of CHEU to CHU.

## Methods

### Setting and design

This longitudinal cohort study includes children followed from September 2013 to March 2020 at Tygerberg Hospital, a public academic and referral centre in Cape Town, South Africa. We recruited three cohorts: CHIV, CHEU and CHU. CHIV were previously in the Children with HIV Early antiRetroviral (CHER) and IMPAACT 1060 clinical trials^8 15 16^ along with CHEU and CHU controls from the same communities. Briefly, the CHER trial compared early time limited ART to deferred ART in infants with perinatal HIV 6–12 weeks of age. First-line ART was lopinavir–ritonavir, zidovudine and lamivudine. The IMPAACT P1060 trial compared nevirapine to lopinavir–ritonavir as a third antiretroviral, with zidovudine and lamivudine in CHIV between 6 months and 3 years of age.

We previously described the initial recruitment, demographic data and results of first pulmonary function test (PFT) performed by these children.^6^

### Inclusion and exclusion criteria

Children were included if they successfully performed at least one lung function test during the study time frame. To minimise the effect of race on pulmonary function outcomes, only black African children were included in this study (online supplemental table 1). PFT outcomes were excluded if the test did not meet European Respiratory Society (ERS), American Thoracic Society (ATS) acceptability criteria.^17–19^

10.1136/thorax-2023-220197.supp1 Supplementary data

### Exposure

The primary comparison was between CHIV and CHU, with a secondary comparison between CHEU and CHU. We chose CHU as the control group in both comparisons as they are unaffected by HIV, therefore, representing the most accurate reflection of the effect of perinatally HIV infection and exposure without infection on lung function in this population.

### Pulmonary function testing

Participants underwent spirometry, body plethysmography and single-breath diffusing capacity, in accordance with American Thoracic Society (ATS) and European Respiratory Society (ERS) guidelines as previously described.^6 17–19^ Briefly, forced maximal expiratory manoeuvres were used to measure forced expiratory volume in 1 s (FEV_1_), forced vital capacity (FVC) and to calculate the FEV_1_/FVC ratio. The best of a minimum of three technically acceptable manoeuvres is reported. Lung volumes and capacities, including total lung capacity (TLC), residual volume (RV), functional residual capacity and vital capacity (VC_pleth_) were examined by body plethysmography. Additionally, we calculated the RV proportion of TLC (RV/TLC). Single breath diffusing capacity was performed to investigate the diffusing capacity of the lung for carbon monoxide (DL_CO_) and to evaluate alveolar volume (V_A_). For plethysmography and diffusing capacity, the average of a minimum of two technically acceptable manoeuvres is reported. Therefore, a total of three spirometry (FVC, FEV_1_, FEV_1_/FVC), four plethysmography (TLC, RV, VC_pleth_, RV/TLC) and two diffusion variables (DL_CO_ and V_A_) were assessed as outcome measures.

To account for the influence of age, height and sex on pulmonary function, all PFT outcomes were converted to z-scores using the Global Lung Initiative (GLI) reference data and software. Spirometry outcome Z-scores (FVC, FEV_1_, FEV_1_/FVC) were calculated using the ‘other’ reference population, which has been shown to best fit the South African population.^20^ Plethysmography (TLC, RV, TLC/RV, VC_pleth_), and diffusing capacity (DL_CO_, VA) Z-scores were calculated with the GLI Caucasian reference population as no ‘other’ reference population is currently available.^21 22^

PFT was performed annually (CHER cohort) and biennially (P1060 cohort), however due to missed appointments, limited PFT facilities, varying ages at time of exit of the parent trial and lost to follow-up, participants have a varying number of PFT performed.

### Confounders and interactions

Confounders were selected using a conceptual causal inference approach, represented by a directed acyclic graph (online supplemental figure 1). Confounders considered included: sex; stature (represented by height, as a continuous variable), age (continuous variable, meancentred to aid interpretation), weight (continuous variable), body mass index (BMI) z-score, pubertal progression—reflected by Tanner stage and smoking (exposure) by urine cotinine levels. BMI-for-age z-scores were used to eliminate collinearity between weight and height, with z-scores calculated using WHO reference equations.^23^ Although GLI z-scores already adjust for age, height and sex, these variables were considered potential confounders, given that the reference population z-scores may not adequately reflect our population.

Urinary cotinine concentration was used as a biomarker for tobacco smoke exposure and was corrected for urine creatinine concentration. Urine cotinine was divided into four groups: <10 ng/mL (non-smoker), 11–30 ng/mL (exposure to secondhand smoke), 30–100 ng/mL (experimental smoker) and ≥100 ng/mL (active smoker). Due to limited data availability, only one cotinine measurement was available for each child, taken at approximately 11 years old. Interaction effects considered for inclusion were age by height, HIV group by age, and sex by HIV group.

### Statistical analysis

We compared baseline characteristics between groups using the χ^2^ test for categorical variables and the F-test for continuous variables. Age, height, weight, BMI-z score, Tanner stage are evaluated at first PFT. Descriptive analyses included graphs of the mean outcome by children’s age and HIV group.

Tanner staging had 48% missingness, therefore, children <8 years old were assigned Tanner stage 1 with children spending a maximum of 2 years in any one stage.^24^ This reduced missingness to 17%. We further used multiple imputation by chained equations using the multivariate imputation by chained equations (mice) in R package and the 21.pan imputation method, given repeated measures spaced unevenly over time.^25 26^ Covariates included in the multiple imputation were sex, HIV group, age, height, weight and Tanner stage. Tanner stage required postprocessing following multiple imputation including: squeezing imputed values to an integer between 1 and 5, squeezing imputed values to values between recorded Tanner stages, and modifying imputed values using to ensure no decreases over time. All clinic visits were included in the imputation model (n=4588), which was subsequently limited to those with at least one lung function outcome (n=946) as part of the mixed effects model and pooled results. Pooled results from 10 imputed datasets are calculated using the broom.mixed package.

Each PFT was modelled as a function of group and child’s age using linear mixed effects models with child-specific random effects. The best among three covariance models: random intercept only, random intercept and random height slope and random intercept and random age slope was chosen using the Akaike information criterion. Significance was declared at level α=0.05.

The mean model included HIV group (three levels), sex and age (continuous), as variables of interest. Interaction effects were included if significant at 0.05 level. Potential confounders were included if significant at 0.20 level using the multivariate Wald test in backward model selection.^27 28^ The final model was fitted using restricted maximum likelihood. Similar models were built for all PFT outcomes, and only significant confounders (p<0.20) were included in each model. Reported CIs and p values are based on the univariate or multivariate Wald test. Statistical analysis was performed using RStudio.

## Results

### Characteristics of children

A total of 328 participants (80 CHU, 126 CHEU, 122 CHIV) successfully performed at least one PFT and were included in this study. Children had PFT measurements between the ages of 6.6 and 15.6 years old.

At time of the first PFT measurement (table 1), CHIV were younger than CHEU and CHU and were shorter in height. The median (IQR) difference between first and last successfully completed PFT was 3.0 (1.9, 5.0) years among CHIV, 0.6 (0, 2.0) years among CHEU and 2.0 (0, 2.3) years among CHU. Online supplemental figure 2 shows the proportion of CHIV with viral suppression over time (mean 91% children virally suppressed).

**Table 1:** Baseline characteristics of the cohort, by HIV group

	CHIV (n=122)	CHEU (n=126)	CHU (n=80)	P value
Age at entry (year)—median (IQR)	8.38 (7.62–8.81)	8.84 (7.80–10.58)	8.77 (8.03–10.36)	<0.001
Sex:				
Male, n (%)	55 (45%)	62 (49%)	44 (55%)	0.386
Female, n (%)	67 (55%)	64 (51%)	36 (45%)	
Height at entry (cm)—median (IQR)	124 (119–129)	130 (124–138)	129 (124–139)	<0.001
Height for age z-score at entry—mean (SD)	−0.83 (0.99)	−0.54 (1.11)	−0.41 (1.19)	0.017
Weight at entry (kg)—median (IQR)	24.3 (22.1–26.9)	27.5 (23.9–33.1)	28.1 (24.1–35.3)	<0.001
BMI at entry—median (IQR)	15.76 (14.97–17.07)	16.25 (15.29–17.58)	16.53 (15.34–18.41)	0.015
BMI z-score at entry—mean (SD)	0.05 (0.93)	0.15 (1.10)	0.38 (1.06)	0.088
Tanner stage at entry:				
Tanner stage 1/2, n (%)	111 (91)	98 (77)	54 (68)	0.086
Tanner stages 3–5, n (%)	1 (1)	6 (5)	4 (5)	
Tanner stage—missing at entry (%)*	10 (8)	22 (17)	22 (28)	
Cotinine levels (ng/mL)				
0–10 ng/mL, n (%)	82 (67)	67 (53)	47 (59)	0.091
10–30 ng/mL, n (%)	22 (18)	39 (31)	15 (19)	
30–100 ng/mL, n (%)	14 (11)	12 (10)	14 (18)	
≥100 ng/mL, n (%)	2 (2)	3 (2)	3 (4)	
Cotinine group—missing, n (%)*	2 (2)	5 (4)	1 (1)	
Antiretroviral drugs				
Zidovudine, n (%)	122 (100)	NA	NA	NA
Lamivudine, n (%)	122 (100)	NA	NA	
Lopinavir/r, n (%)	103 (84)	NA	NA	
Nevirapine, n (%)	21 (17)	NA	NA	
Efavirenz, n (%)	2 (3)	NA	NA	
Median age at ART, years initiation (months)—median (IQR)	4.0 (1.8–8.3)			

Age, height, weight, BMI z-score at entry at first successfully PFT. Median (IQR) or n (%), and p values (χ^2^/ F-test) are reported.

*Missing values are not included in the inferential analysis.

ART, antiretroviral therapy; BMI, body mass index; CHEU, Children who are HIV exposed uninfected; CHIV, children with perinatally acquired HIV; CHU, children who are HIV unexposed; NA, not available; PFT, pulmonary function testing.

At baseline lung function testing, there was no significant difference in spirometry, plethysmography or diffusing capacity outcomes between CHU, CHEU, CHIV (online supplemental table 2).

### Spirometry (FEV_1_, FVC, FEV_1_/FVC)

**Figure 1:**
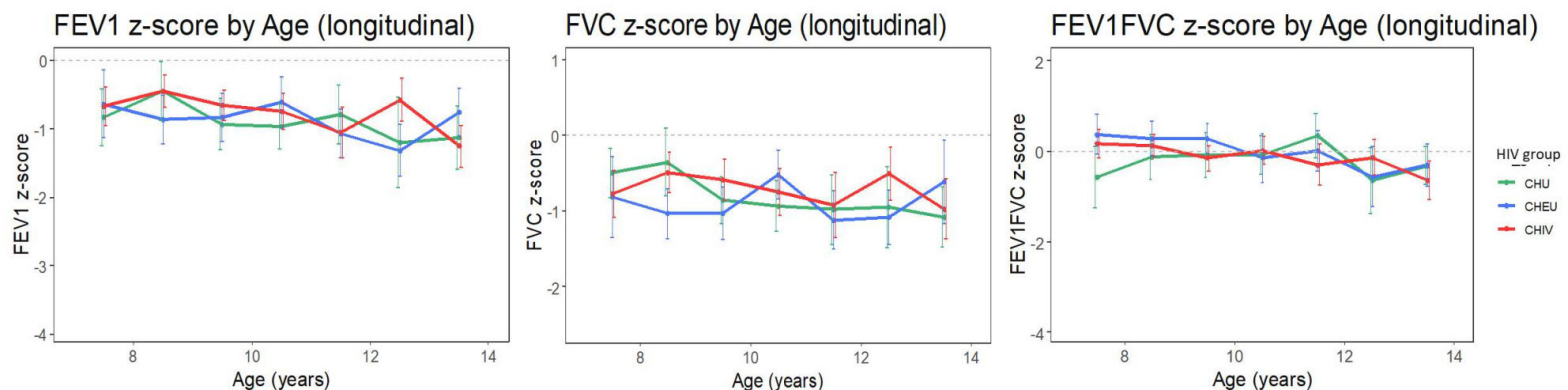
Spirometry outcome tracking though childhood in children with perinatally acquired HIV (CHIV), children who are HIV exposed uninfected (CHEU) and children who are HIV unexposed (CHU). Unadjusted means (with 95% CI) of spirometry measures by HIV group. Means are calculated for each yearly age band. The light grey dashed line represents z=0. FVC, forced vital capacity; FEV_1_, forced expiratory volume in 1 s.

The unadjusted progression of FEV_1_, FVC and FEV_1_/FVC remained relatively flat over time (figure 1). For all study groups, FEV_1_ and FVC tracked near a z-score of −1 with the FEV_1_/FVC ratio tracking near a z-score of 0 over time.

Longitudinal single-predictor analysis (online supplemental tables 3–5) did not find a significant difference over time in Z-scores of FEV_1_, FVC or FEV_1_/FVC between CHIV and CHU, and between CHEU and CHU. Sex, age and height were found to be significantly associated with FEV_1_, FVC or FEV_1_/FVC Z-scores (p<0.05).

In multipredictor analysis (online supplemental tables 3–5), after adjusting for relevant confounders (p<0.20) in each spirometry outcome, no significant difference in FEV_1_, FVC or FEV_1_/FVC Z-score was found between CHIV and CHU, and between CHEU and CHU (p>0.05).

### Plethysmography (TLC, RV, RV/TLC, VC_pleth_)

**Figure 2:**
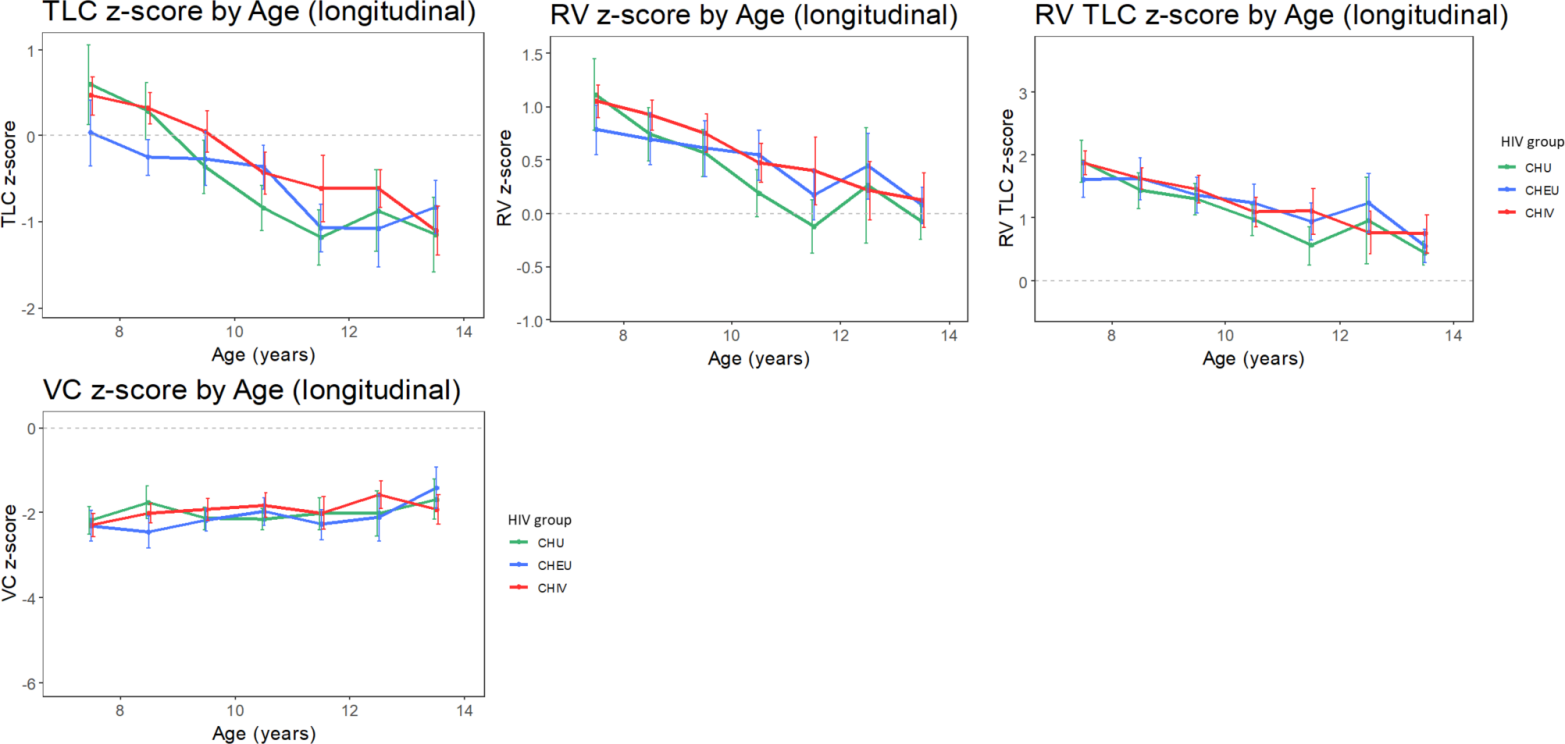
Plethysmography outcome tracking though childhood in children with perinatally acquired HIV (CHIV), children who are HIV exposed uninfected (CHEU) and children who are HIV unexposed (CHU). Unadjusted means (with 95% CI) of plethysmography measures by HIV group. Means are calculated for each yearly age band. The light grey dashed line represents z=0. RV TLC, ratio of RV/TLC; RV, residual volume; TLC, total lung capacity; VC_pleth_, slow vital capacity.

Unadjusted plethysmography Z-scores were not stable over time with a marked decrease in TLC, RV, RV/TLC Z-score in all groups over time (figure 2). However, VC_pleth_ was stable and tracked along Z-score of −2 with time.

Single-predictor analysis (online supplemental table 6–9) showed TLC, RV and RV/TLC z-scores to be significantly higher in CHIV compared with CHU. No significant difference was found between CHEU and CHU. There was no significant difference in VC_pleth_ z-score between HIV groups. Age, height, weight, BMI z-score and Tanner stage showed a significant association with plethysmography z-scores.

In multipredictor analysis for TLC z-score (online supplemental table 6), no significant difference was found between CHIV and CHU. However, in the comparison between CHEU and CHU, the interaction between HIV group and age was significant (p=0.008), with CHEU starting at a lower TLC-z-score compared with CHU at age 7, with less decline over time, see also online supplemental figure 3.

The mixed effects model for RV z-score (online supplemental table 7) revealed that, after adjusting for sex, age and height, the mean RV z-score ratio was 17% greater in CHIV than CHU (95% CI 1% to 33% p=0.042). There was no significant difference between CHEU and CHU.

Multipredictor analysis for RV/TLC and VC_pleth_ z-score revealed no significant difference between CHIV and CHU, and CHEU and CHU, after adjusting for relevant confounders (online supplemental tables 8 and 9).

### Diffusing capacity (DL_CO_, V_A_)

**Figure 3:**
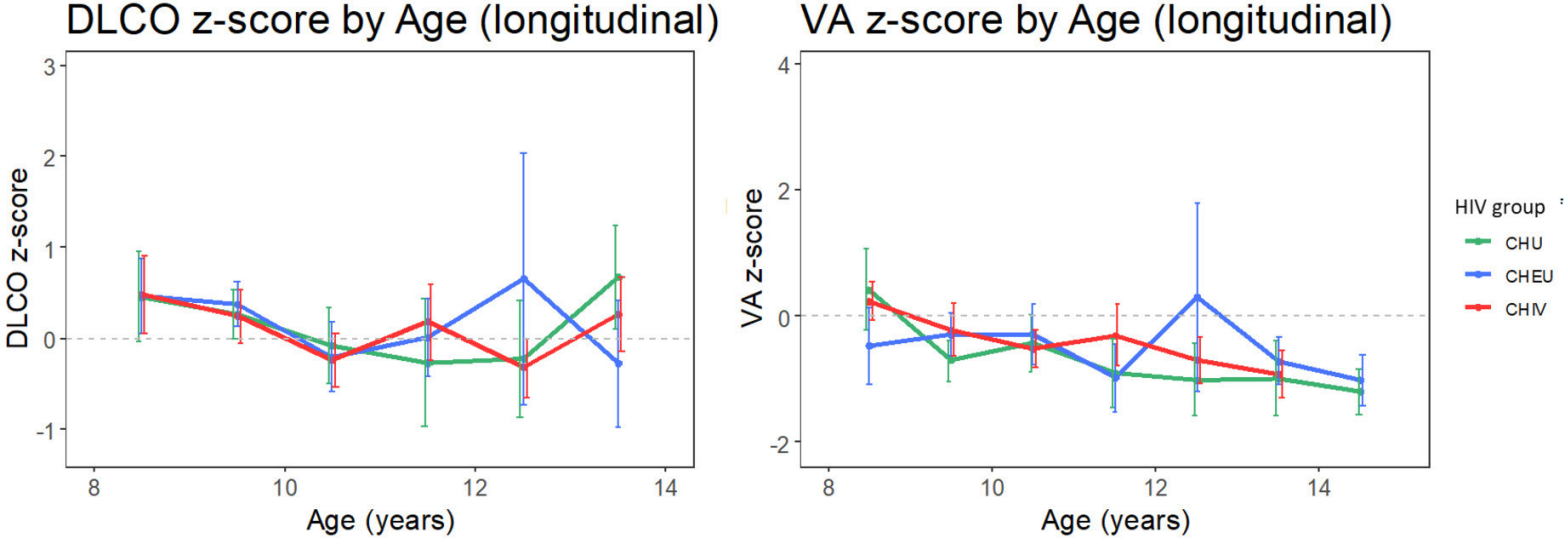
Diffusing capacity tracking through childhood in children with perinatally acquired HIV (CHIV), children who are HIV exposed uninfected (CHEU) and children who are HIV unexposed (CHU). Unadjusted means (with 95% CI) of diffusing capacity measures by HIV group. Means are calculated for each yearly age band. D_LCO_, diffusing capacity of the lung for carbon monoxide; V_A_, alveolar volume by helium dilution. The light grey dashed line represents z=0.

Unadjusted diffusing capacity outcome (D_LCO_ and V_A_) Z-scores remained stable over time and tracked near Z=0 (figure 3).

In single-predictor analysis, no significant difference in D_LCO_ or V_A_ z-score was found between CHIV and CHU, and between CHEU and CHU (online supplemental tables 10 and 11). However, age, height, weight and Tanner stage were found to be significantly associated with a diffusing capacity measure (DL_CO_ or V_A_).

In multipredictor analysis for D_LCO_ (online supplemental table 10), no significant difference in was found between CHIV and CHU, and between CHEU and CHU. In multipredictor analysis for V_A_ (online supplemental table 11), the interaction between and HIV group and sex was significant, indicating that the difference in V_A_ z-score between CHIV and CHU and CHEU and CHU differs by sex (online supplemental figure 4) depicts this interaction.

## Discussion

In this study, we demonstrate that in CHIV early initiation of ART within the first 3 years of life (median 4 months), has lasting benefit to lung function with lung function trajectory being largely similar to CHU throughout childhood. Our findings indicate that the early initiation of ART may attenuate the decline of lung function associated with perinatal HIV infection and HIV-associated lung disease. Furthermore, we demonstrate that lung function in children unexposed to HIV did not differ significantly, and therefore, suggesting that lung development is not substantially disrupted by perinatal HIV-exposure.

While the findings of our study are promising, there remains concern regarding small airway disease in CHIV. Plethysmography demonstrates that RV is higher in CHIV, which may indicate subtle small airway disease not identified by spirometry. The higher RV in CHIV is not accompanied with increased RV/TLC. Therefore, our findings, while suggestive of small airway disease, do not provide definitive proof of air-trapping. Given the lack of abnormal spirometry data, it is unlikely that these findings are due to significant bullae or lung cysts. These findings are in keeping with studies in non-infectious paediatric lung disease, which demonstrate that spirometry may fail to detect early small airway disease in children.^29–31^ Therefore, additional lung function tests including, plethysmography, multiple breath washout and forced oscillation should be considered in CHIV, as these PFTs have demonstrated an improved ability to identify early small airway and structural lung disease in children.^29–31^

Despite the significance of childhood HIV-associated lung disease, there is a paucity of lung function data in CHIV. Most studies have focused on spirometry with few employing plethysmography, multiple breath washout or forced oscillation to examine small airway function. Our study highlights that plethysmography can identify signals of small airway disease not found by spirometry. It is noteworthy that all groups in our study have a RV z-score above the mean until roughly 12 years of age. Considering that VC_pleth_ remains stable over time and the decrease in TLC z-score relates to diminishing RV and RV/TLC, a reasonable hypothesis is that prenatal and postnatal exposures result in small airway injury leading to air-trapping which improves with lung and airway calibre growth during childhood. The South African low-income population, from which our study recruited participants, is exposed to multiple socioeconomic and environmental factors known to disrupt lung health and injure small airways. Environmental exposures, including antenatal and postnatal exposure to air pollution and smoking, can result in airflow limitation but not a reduction of lung volume as approximated by FVC and multiple breath wash-out.^4 32^ As all participants were recruited from a similar socioeconomic and geographic population, it is possible that all participants had early in life small airway disease, which was more pronounced in CHIV.

An alternative explanation is that the GLI reference populations and equations do not adequately represent our study population. Spirometry reference data of the GLI ‘other’ population has been shown to have a reasonable fit to South African children.^20^ However, in our study sex, age and height were found to be significantly associated with FEV_1_, FVC or FEV_1_/FVC Z-scores indicating that the GLI reference equation may not fully take these variables into account for South African children. Similarly, multiple demographic characteristics including age, height and weight were significantly associated with plethysmography and diffusing capacity Z-scores. It is noteworthy that to date only ‘Caucasian’ reference data are available for plethysmography and diffusing capacity. The determinants of lung function are complicated, involving the interaction of multiple factors, including anthropometry and body proportion (specifically thoracic size), socioeconomic status, and environmental exposures. These factors are not fully represented in the current GLI reference equation and could explain the differences observed in the plethysmography outcomes in our study population and the GLI reference population.^33^

The findings of our study are relevant in the current context of HIV care. Early ART initiation has been shown to benefit survival and neurocognitive outcome in CHIV. However, data on the effect of early ART on HIV-associated lung disease and childhood lung function are scarce. Available lung function evidence following the early initiation of ART in CHIV is encouraging. Though cross-sectional, these studies indicate that early ART may attenuate the effect of HIV-associated lung disease on childhood lung function.^6 9^ Longitudinal studies in the modern era of early ART are limited and it was previously unclear whether the lung function benefit offered by early ART extends beyond childhood.

In contrast to the few studies examining early ART initiation, most previous lung function studies of CHIV have examined lung function in children with ART initiation after the first few years of life. These studies have clearly demonstrated that if the diagnosis of HIV infection or treatment with ART is delayed, childhood lung function is impaired. Though longitudinal data are limited, these children have decreased lung function throughout childhood with lung function trajectory tracking lower that HIV-uninfected children.^11^ The primary abnormality of HIV-associated lung disease is that of air-flow limitation, although restrictive defects are seen. Airflow limitation is fixed with limited improvement during childhood lung growth indicating that once established, small airway injury in HIV-associated lung disease is irreversible. The underlying mechanism has not been clearly demonstrated and is thought to be a combination of recurrent infection and dysregulated inflammatory response.

Longitudinal lung function studies of other childhood small airway disease have demonstrated the benefit of early therapeutic intervention. Comprehensive studies performed in cystic fibrosis, a genetic condition leading to small airway disease starting early in life, have investigated the effect of early pulmonary therapy on lung structure and function. Despite different pathophysiology, cystic fibrosis studies demonstrate that early intervention can limit airway inflammation and injury leading to symptom reduction, improved function and less structural lung disease.^34 35^

It is becoming increasingly evident that insults to childhood lung development play an important role in lung disease throughout life. Longitudinal studies have focused on non-infectious lung diseases, including asthma, chronic neonatal lung disease and early life exposure to environmental pollutants and tobacco smoke. These studies have clearly demonstrated that early life factors play a significant role in determining lung function later in life. Furthermore, diminished lung function as a child leads to poor adult lung function and increased risk of COPD.^12^

Our study provides the first available evidence that early ART initiation may mitigate the lung function impairment coupled with HIV-associated lung disease and have lasting benefit throughout childhood. It remains to be seen whether the childhood lung function advantage of early ART initiation extends into adulthood.

### Strengths, weakness

A major strength of this study is the large number of children who performed extensive PFT during childhood. Moreover, CHEU and CHU were included as control populations to allow for the examination of the influence of antenatal HIV exposure without infection in addition to that of HIV infection on childhood lung function. The addition of plethysmography allowed this study to identify signs of small airway disease not found by spirometry.

Limitations include the heterogenicity of the CHIV population which prevents subgroup analysis, the lower rate of follow-up PFT in the CHEU cohort and the relative low number of diffusing capacity tests performed. A history of pulmonary tuberculosis or pneumonia was not accounted for, and the current study did not include lung imaging to ascertain whether the improvement of lung function is linked to less structural lung disease.

## Conclusion

Early ART initiation can mitigate the loss of lung function associated with perinatally acquired HIV and has lasting benefit throughout childhood and potentially into adulthood. Furthermore, perinatal HIV exposure without infection does not appear to disrupt lung function trajectory. There is a need for reference data that reflect the diverse population with multiple and mixed ancestries in South Africa and other African settings.

## Data Availability

Data are available on reasonable request. Sharing of deidentified participant spirometry, plethysmography and diffusion capacity data is subject to approval of an signed data sharing agreement and participation of the first and senior authors in collaborative statistical analysis, data interpretation and manuscript drafting. Data will be made available following the publication of this manuscript, provided the above criteria are met. Requests to access this data should be made to agie@sun.ac.za, steven.innes@hiv-research.org.za
