# Supplementary material for: Lung function tracking in children with perinatally acquired HIV following early antiretroviral therapy initiation
Source: Thorax. 2023 Jul 21;78(12):1233–9. doi: 10.1136/thorax-2023-220197 (PMC10715541; doi:10.1136/thorax-2023-220197)
Supplement: Supplementary data [file thorax-2023-220197supp001.pdf]

Supplementary data:

Supplementary data
For all tables and figures the following abbreviations are used;

CHIV: Children living with perinatally-acquired HIV
CHEU: Children who are HIV exposed uninfected
CHU: Children who are HIV unexposed

Supplementary figure 1: Directed Acyclic Graph (DAG) diagram to determine potential confounders.

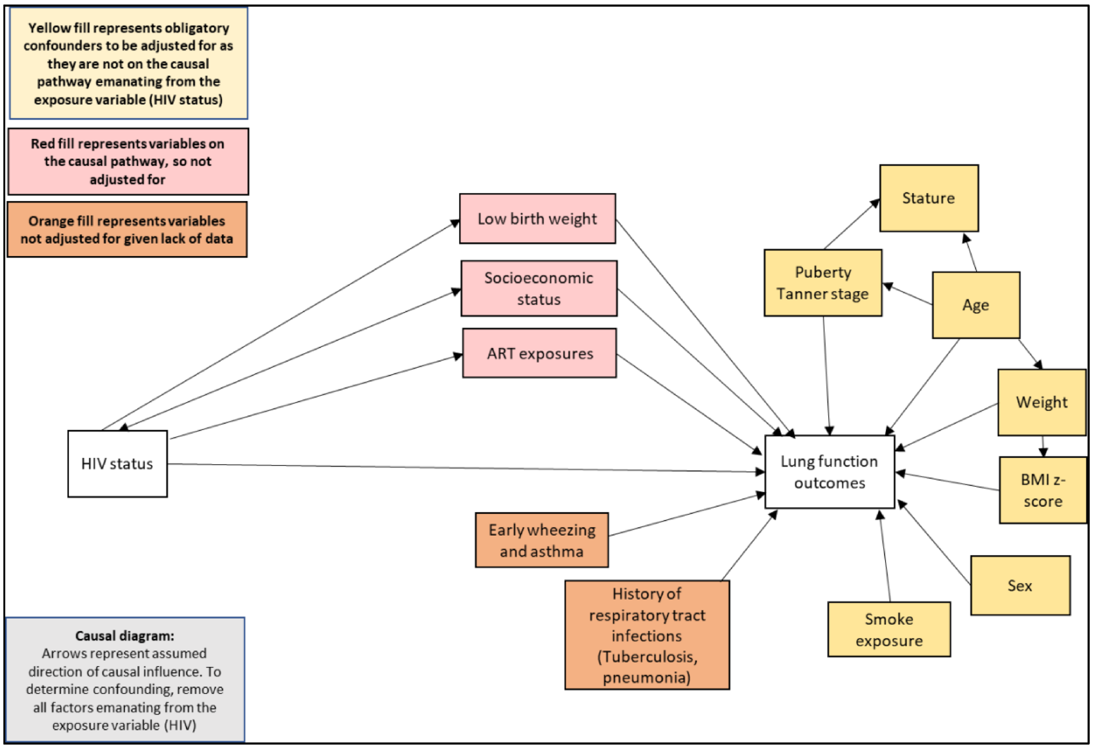

Gie A, *et al. Thorax* 2023; 78:1233–1239. doi: 10.1136/thorax-2023-220197

Supplementary Figure 2: Proportion of CHIV with viral suppression by age band.

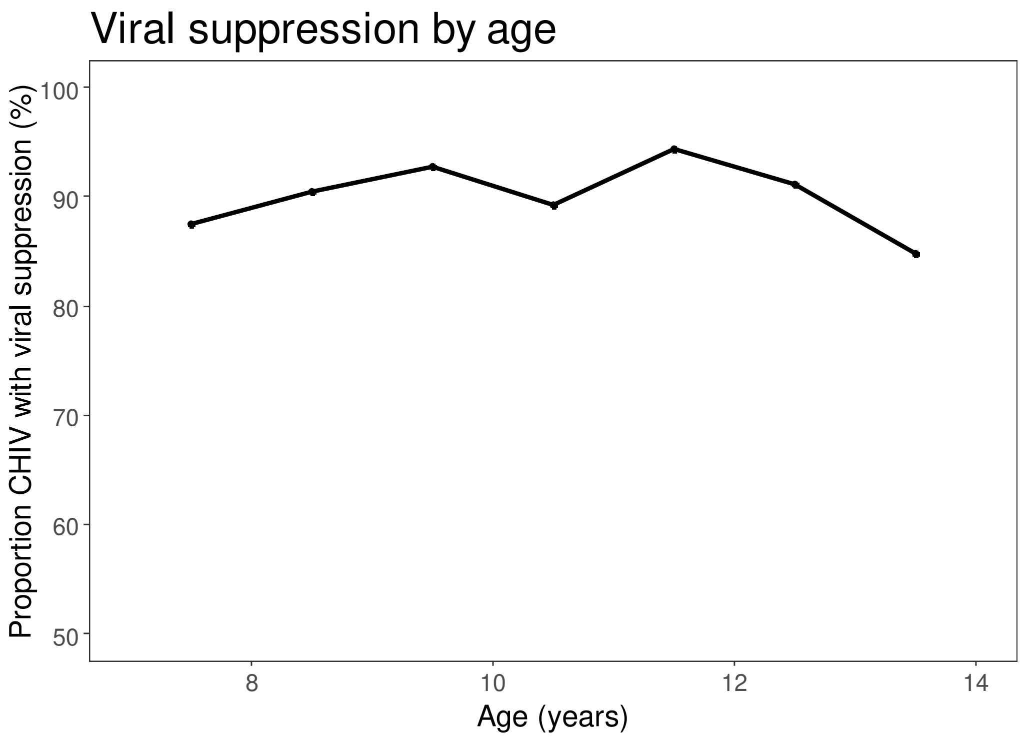

CHIV: Children living with perinatally-acquired HIV. HIV viral suppression was defined as an HIV viral load of <400 copies.mL^{-1} . A child is defined as suppressed if their viral load is undetectable for all viral load measurements in that age band.

Gie A, *et al. Thorax* 2023; 78:1233–1239. doi: 10.1136/thorax-2023-220197

Supplementary Figure 3: Effect display for the interaction of HIV group and age in the linear mixed model for TLC z-score

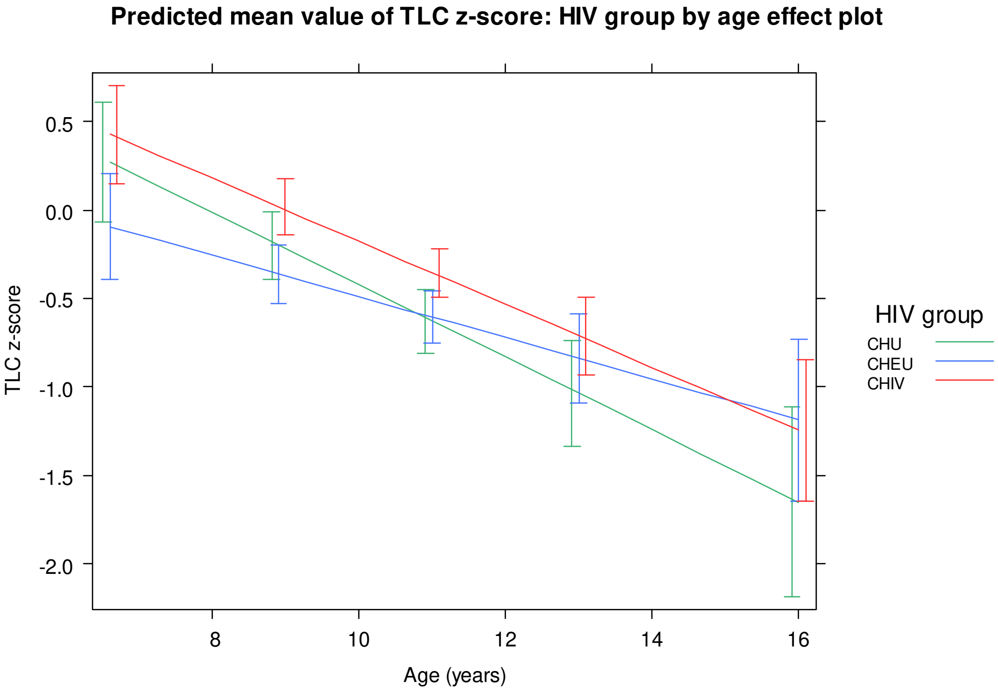

This figure is a graphical depiction of the terms from the linear mixed model, after adjusting for age, height, weight, BMI z-score, Tanner stage, and the interaction between HIV group and age. The figure has been constructed using the Effects package in R, and therefore the analysis only reflects the fixed effects of the model (not random effects) and assumes that other variables in the model are constant and take on their average values. The graphs are constructed using one random dataset from a set of 10 datasets constructed using multiple imputation, and the results do not differ across imputations. 95% Confidence intervals are shown, with standard errors computed from the covariance matrix of the fitted regression coefficients.

Gie A, *et al. Thorax* 2023; 78:1233–1239. doi: 10.1136/thorax-2023-220197

Supplementary Figure 4: Effect display for the interaction of HIV group and gender in the linear mixed model for VA z-score

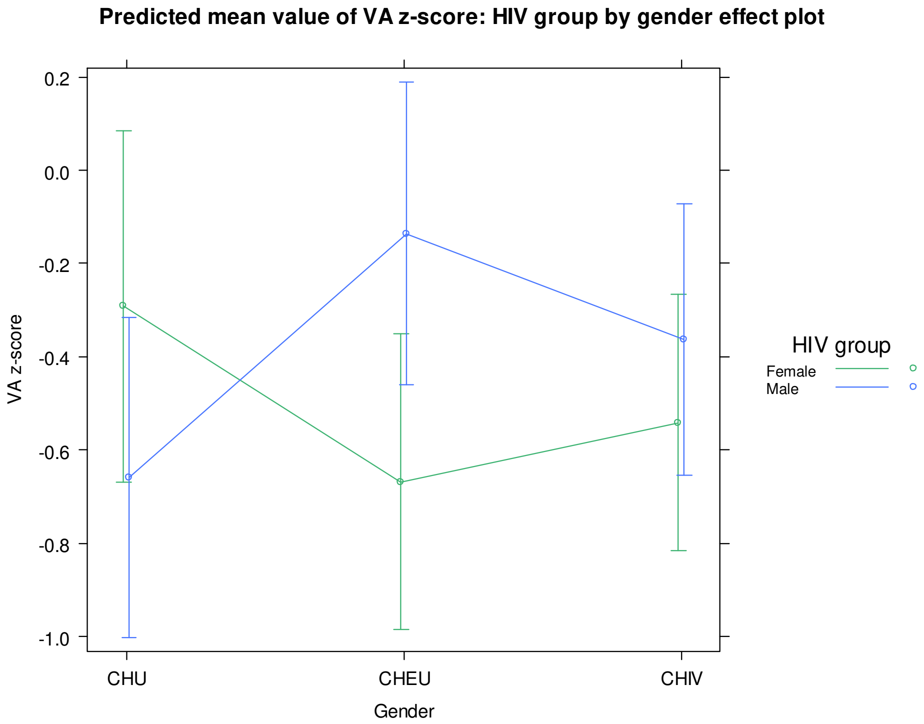

This figure is a graphical depiction of the terms from the linear mixed model, after adjusting for gender, height, weight, cotinine group, and the interaction between HIV group and gender. The figure has been constructed using the Effects package in R, and therefore the analysis only reflects the fixed effects of the model (not random effects) and assumes that other variables in the model are constant and take on their average values. 95% Confidence intervals are shown, with standard errors computed from the covariance matrix of the fitted regression coefficients.

Gie A, *et al. Thorax* 2023; 78:1233–1239. doi: 10.1136/thorax-2023-220197

Supplementary table 1: Ethnicity analysis

	**Black children only**	**Mixed children only**	**Combined**
FEV1			
CHIV	0·23 (-0·04 to 0·50)	-0.44 (-1.07 to 0·20)	-0·01 (-0·37 to 0·35)[1]
CHEU	0·03 (-0·25 to 0·30)	-0·44 (-1·12 to 0·24)	-0·24 (-0·63 to 0·13)
FVC			
CHIV	0·15 (-0·13 to 0·43)	-0·20 (-0·84 to 0·44)	0·05 (-0·20 to 0·31)
CHEU	-0·10 (-0·39 to 0·19)	-0·40 (-1·08 to 0·29)	-0·21 (-0·47 to 0·06)
FEV1FVC			
CHIV	0·14 (-0·16 to 0·43)	-0·48 (-1·01 to 0·06)	-0·00 (-0·24 to 0·24)
CHEU	0·26 (-0·04 to 0·55)	-0·20 (-0·77 to 0·38)	0·11 (-0·14 to 0·36)
DLCO			
CHIV	0·03 (-0·24 to 0·31)	-0·26 (-1·07 to 0·55)	0·03 (-0·23 to 0·28)
CHEU	0·03 (-0·26 to 0·32)	-0·31 (-1·06-0·44)	0·08 (-0·19 to 0·36)
VA			
CHIV	-0·26 (-0·72 to -0·19)[2]	-0·60 (-1·24 to 0·04)	-0·30 (-0·65 to 0·06)[2]
CHEU	**-0·42 (-0·89 to -0·12)**	-0·21 (-0·82 to 0·41)	**-0·47 (-0·84 to -0·10)**
TLC			
CHIV	0·11 (-0·94 to 0·72)[3]	0·09 (-0·29 to 0·48)	**0·21 (0·03 to 0·40)**
CHEU	**-1·02 (-1·93 to -0·12)**	-0·14 (-0·57 to 0·30)	-0·06(-0·25 to 0·12)
RV			
CHIV	**0·17 (0·01 to 0·33)**	0·14 (-0·19 to 0·47)	**0·19 (0·06 to 0·32)**
CHEU	0·09 (-0·08 to 0·25)	-0·08 (-0·47 to 0·30)	0·10 (-0·04 to 0·24)
RV/TLC			
CHIV	0·11 (-0·09 to 0·31)	0·21 (-0·21 to 0·62)	0·11 (-0·07 to 0·29)
CHEU	0·14 (-0·07 to 0·35)	-0·06 (-0·53 to 0·41)	0·14 (-0·05 to 0·32)

Association between each HIV group and each lung function outcome using linear mixed effects models. The models are based on three datasets: Black children only; Mixed race children only; and the combined dataset. All results are after adjusting for relevant confounders (p<0.20) and after multiple imputation. Only the results for the HIV group variable are displayed, with results showing the mean difference (with 95% CI) in z-score between the exposed group (CHIV or CHEU) and the unexposed group, CHU. Results highlighted in bold indicate significant results.

Gie A, *et al. Thorax* 2023; 78:1233–1239. doi: 10.1136/thorax-2023-220197

1. Interaction between HIV group and ethnicity, and interaction between HIV group and gender significant
2. Interaction between HIV group and gender significant
3. Interaction between HIV group and age significant

Supplementary Table 2: Baseline lung function

	CHIV (n = 122)	CHEU (n = 126)	CHU (n = 80)	p-value
Spirometry				
FEV1 z-score - mean (SD)	-0.64 (1.06)	-0.83 (1.14)	-0.85 (1.03)	0.285
FVC z-score - mean (SD)	-0.76 (1.16)	-1.00 (1.19)	-0.87 (0.99)	0.254
FEV_1/FVC z-score - mean (SD)	0.22 (1.08)	0.30 (1.22)	0.00 (1.26)	0.21
Plethysmography				
TLC z-score - mean (SD)	0.23 (0.97)	-0.37 (0.94)	-0.26 (1.03)	<0.001
RV z-score - mean (SD)	0.94 (0.65)	0.66 (0.74)	0.60 (0.75)	0.001
RV/TLC z-score - mean (SD)	1.73 (0.81)	1.50 (0.94)	1.39 (0.84)	0.02
VC z-score - mean (SD)	-2.18 (1.04)	-2.37 (1.04)	-2.20 (0.86)	0.297
Diffusing capacity:				
DLCO z-score - mean (SD)	0.27 (0.93)	0.13 (1.28)	0.15 (0.99)	0.664
V_A z-score - mean (SD)	-0.25 (0.92)	-0.41 (1.24)	-0.54 (1.07)	0.261

Baseline lung function as at the first successful spirometry, plethysmography, and diffusing capacity maneuver. Mean (SD), and p-values (F-test) are reported.

Gie A, *et al. Thorax* 2023; 78:1233–1239. doi: 10.1136/thorax-2023-220197

Supplementary Table 3: FEV_1

Association between each HIV group and FEV_1 z-score using linear mixed effects model.

Unadjusted estimates show single-predictor analysis of each variable on FEV_1 z-score.

Adjusted results show the mean difference (with 95% CI) in FEV_1 z-score between the exposed group (CHIV/CHEU) and the unexposed group, CHU.

Predictors	Unadjusted Estimate (95% CI)	p-value	Adjusted Estimate (95% CI)	p-value
CHIV (vs CHUU)	0.18 (-0.1 to 0.45)	0.204	0.23 (-0.04 to 0.50)	0.092
CHEU (vs CHUU)	0.02 (-0.26 to 0.3)	0.869	0.03 (-0.25 to 0.30)	0.851
Sex: Male	0.18 (-0.04 to 0.39)	0.103	0.2 (-0.01 to 0.41)	0.063
Age (per year)	-0.06 (-0.09 to -0.03)	<0.001	-0.05 (-0.08 to -0.02)	0.003
Height (per 1cm increase)	-0.01 (-0.01 to 0)	0.004	-	-
Weight (per 1kg increase)	-0.01 (-0.01 to 0)	0.092	-	-
BMI z-score	0.09 (0.01 to 0.18)	0.028	0.1 (0.02 to 0.18)	0.021
Cotinine group: <10ng/mL (reference)	-	-	-	-
11-30ng/mL	-0.06 (-0.32 to 0.2)	0.641	-	-
30-100 ng/mL	-0.11 (-0.44 to 0.23)	0.535	-	-
>100 ng/mL	-0.47 (-1.14 to 0.21)	0.173	-	-
Tanner stage 1: reference	-	-	-	-
Tanner stage (II vs. I)	-0.27 (-0.45 to -0.09)	0.003	-	-
Tanner stage (III vs. I)	-0.08 (-0.31 to 0.15)	0..487	-	-
Tanner stage(IV vs. I)	-0.17 (-0.68 to 0.35)	0.526	-	-
Tanner stage (V vs. I)	-0.76 (-2.81 to 1.29)	0.465	-	-

Results show the adjusted differences in z-scores between HIV groups. The model with random intercept only was optimal. Multi-predictor analysis is shown after adjusting for sex, age and BMI z-score.

CHU: Children who are HIV unexposed; CHEU: Children who are HIV exposed uninfected; CHIV: Children with perinatally-acquired HIV

Gie A, *et al. Thorax* 2023; 78:1233–1239. doi: 10.1136/thorax-2023-220197

Supplementary Table 4: FVC

Association between each HIV group and FVC z-score using linear mixed effects model.

Unadjusted estimates show single-predictor analysis of each variable on FVC z-score.

Adjusted results show the mean difference (with 95% CI) in FVC z-score between the exposed group (CHIV/CHEU) and the unexposed group, CHU.

Predictors	Unadjusted Estimate (95% CI)	p-value	Adjusted Estimate (95% CI)	p-value
CHIV (vs CHUU)	0.13 (-0.15 to 0.42)	0.360	0.15 (-0.13 to 0.43)	0.295
CHEU (vs CHUU)	-0.10 (-0.39 to 0.19)	0.491	-0.1 (-0.39 to 0.19)	0.495
Sex: Male	0.06 (-0.16 to 0.29)	0.577	-	-
Age (per year)	-0.02 (-0.05 to 0.01)	0.259	0.06 (-0.02 to 0.15)	0.144
Height (per 1cm increase)	0.00 (-0.01 to 0.00)	0.143	-0.01 (-0.03 to 0.00)	0.063
Weight (per 1kg increase)	0.00 (-0.01 to 0.01)	0.718	-	-
BMI z-score	0.13 (0.04 to 0.22)	0.003	0.17 (0.08 to 0.27)	<0.001
Cotinine group: <10ng/mL (reference)	-	-	-	-
11-30ng/mL	-0.16 (-0.43 to 0.11)	0.252	-0.19 (-0.46 to 0.08)	0.103
30-100 ng/mL	-0.04 (-0.39 to 0.31)	0.804	0.01 (-0.34 to 0.37)	
>100 ng/mL	-0.77 (-1.47 to -0.06)	0.033	-0.73 (-1.40 to -0.05)	
Tanner stage 1: reference	-	-	-	-
Tanner stage (II vs. I)	-0.22 (-0.41 to -0.03)	0.026	-	-
Tanner stage (III vs. I)	0.03 (-0.22 to 0.28)	0.827	-	-
Tanner stage(IV vs. I)	0.12 (-0.43 to 0.68)	0.662	-	-
Tanner stage (V vs. I)	-0.85 (-3.05 to 1.35)	0.446	-	-

Results show the adjusted differences in z-scores between HIV groups. The model with random intercept only was optimal. Multi-predictor analysis is shown after adjusting for age, height, BMI z-score and cotinine group.

CHU: Children who are HIV unexposed; CHEU: Children who are HIV exposed uninfected; CHIV: Children living with perinatally-acquired HIV

Gie A, *et al. Thorax* 2023; 78:1233–1239. doi: 10.1136/thorax-2023-220197

Supplementary Table 5: FEV1/FVC z-score

Association between each HIV group and FEV1FVC z-score using linear mixed effects model. Unadjusted estimates show single-predictor analysis of each variable on FEV1FVC z-score. Adjusted results show the mean difference (with 95% CI) in FEV1FVC z-score between the exposed group (CHIV/CHEU) and the unexposed group, CHU.

Predictors	Unadjusted Estimate (95% CI)	p-value	Adjusted Estimate (95% CI)	p-value
CHIV (vs CHUU)	0.09 (-0.21 to 0.39)	0.552	0.14 (-0.16 to 0.43)	0.362
CHEU (vs CHUU)	0.23 (-0.08 to 0.54)	0.141	0.26 (-0.04 to 0.55)	0.092
Sex: Male	0.28 (0.05 to 0.51)	0.018	0.25 (-0.02 to 0.48)	0.033
Age (per year)	-0.09 (-0.13 to -0.05)	<0.001	-0.20 (-0.28 to -0.12)	<0.001
Height (per 1cm increase)	-0.01 (-0.02 to 0)	0.006	0.04 (0.02 to 0.06)	<0.001
Weight (per 1kg increase)	-0.02 (-0.03 to -0.01)	<0.001	-0.03 (-0.05 to -0.01)	0.002
BMI z-score	-0.07 (-0.17 to 0.03)	0.164	-	-
Cotinine group: <10ng/mL (reference)	-	-	-	-
11-30ng/mL	0.13 (-0.16 to 0.42)	0.384	-	-
30-100 ng/mL	-0.12 (-0.49 to 0.25)	0.523	-	-
>100 ng/mL	0.47 (-0.27 to 1.22)	0.212	-	-
Tanner stage 1: reference	-	-	-	-
Tanner stage (II vs. I)	-0.16 (-0.39 to 0.06)	0.159	-	-
Tanner stage (III vs. I)	-0.23 (-0.52 to 0.06)	0.114	-	-
Tanner stage(IV vs. I)	-0.63 (-1.26 to -0.01)	0.045	-	-
Tanner stage (V vs. I)	-0.22 (-2.54 to 2.09)	0.850	-	-

Results show the adjusted differences in z-scores between HIV groups. The model with random intercept only was optimal. Multi-predictor analysis is shown after adjusting for sex, age, height, weight, and BMI z-score.

CHU: Children who are HIV unexposed; CHEU: Children who are HIV exposed uninfected; CHIV: Children living with perinatally-acquired HIV

Gie A, *et al. Thorax* 2023; 78:1233–1239. doi: 10.1136/thorax-2023-220197

Supplementary Table 6: TLC z-score

Association between each HIV group and TLC z-score using linear mixed effects model. Unadjusted estimates show single-predictor analysis of each variable on TLC z-score. Adjusted results show the mean difference (with 95% CI) in TLC z-score between the exposed group (CHIV/CHEU) and the unexposed group, CHU.

Predictors	Unadjusted Estimate (95% CI)	p-value	Adjusted Estimate (95% CI)	p-value
CHIV (vs CHUU)	0.34 (0.12 to 0.57)	0.003	-0.11 (-0.94 to 0.72)	0.794
CHEU (vs CHUU)	-0.06 (-0.29 to 0.18)	0.636	-1.02 (-1.93 to -0.12)	0.026
Sex: Male	0.03 (-0.15 to 0.21)	0.737		
Age (per year, centred at the mean)	-0.27 (-0.29 to -0.24)	<0.001	-0.2 (-0.29 to -0.12)	<0.001
Height (per 1cm increase)	-0.05 (-0.05 to -0.04)	<0.001	-0.04 (-0.05 to -0.02)	<0.001
Weight (per 1kg increase)	-0.04 (-0.05 to -0.04)	<0.001	0.02 (0.01 to 0.03)	<0.001
BMI z-score	0.08 (0.01 to 0.16)	0.032	-	-
Cotinine group: <10ng/mL (reference)	-	-	-	-
11-30ng/mL	-0.04 (-0.27 to 0.18)	0.698	-	-
30-100 ng/mL	-0.08 (-0.37 to 0.21)	0.605	-	-
>100 ng/mL	-0.49 (-1.06 to 0.09)	0.099	-	-
Tanner stage 1: reference	-	-	-	-
Tanner stage (II vs. I)	-1.03 (-1.21 to -0.85)	<0.001	-0.13 (-0.3 to 0.03)	0.017
Tanner stage (III vs. I)	-0.98 (-1.21 to -0.75)	<0.001	0.14 (-0.11 to 0.39)	
Tanner stage(IV vs. I)	-1.08 (-1.57 to -0.59)	<0.001	0.32 (-0.12 to 0.77)	
Tanner stage (V vs. I)	-1.74 (-3.56 to 0.09)	0.062	0.7 (-0.78 to 2.18)	
Interaction: CHIV*age			0.03 (-0.04 to 0.11)	0.056
Interaction: CHEU*age			0.09 (0.01 to 0.18)	

Results show the adjusted differences in z-scores between HIV groups. The model with random intercept and random slope with height was optimal. Multi-predictor analysis is shown after adjusting for age, height, weight, Tanner stage, and the interaction between HIV group and age.

CHU: Children who are HIV unexposed; CHEU: Children who are HIV exposed uninfected; CHIV: Children living with perinatally-acquired HIV

Gie A, *et al. Thorax* 2023; 78:1233–1239. doi: 10.1136/thorax-2023-220197

Supplementary Table 7: RV z-score

Association between each HIV group and RV z-score using linear mixed effects model.

Unadjusted estimates show single-predictor analysis of each variable on RV z-score.

Adjusted results show the mean difference (with 95% CI) in RV z-score between the exposed group (CHIV/CHEU) and the unexposed group, CHU.

Predictors	Unadjusted Estimate (95% CI)	p-value	Adjusted Estimate (95% CI)	p-value
CHIV (vs CHUU)	0.25 (0.09 to 0.41)	0.003	0.17 (0.01 to 0.33)	0.042
CHEU (vs CHUU)	0.09 (-0.08 to 0.26)	0.322	0.09 (-0.08 to 0.25)	0.300
Sex: Male	-0.16 (-0.29 to -0.03)	0.014	-0.16 (-0.28 to -0.03)	0.013
Age (per year)	-0.17 (-0.2 to -0.15)	<0.001	-0.13 (-0.18 to -0.08)	<0.001
Height (per 1cm increase)	-0.03 (-0.03 to -0.02)	<0.001	-0.01 (-0.02 to 0.00)	0.058
Weight (per 1kg increase)	-0.03 (-0.03 to -0.02)	<0.001		
BMI z-score	0 (-0.06 to 0.05)	0.876		
Cotinine group: <10ng/mL (reference)	-	-	-	-
11-30ng/mL	0.1 (-0.06 to 0.26)	0.236		
30-100 ng/mL	-0.03 (-0.24 to 0.18)	0.770		
>100 ng/mL	0.15 (-0.26 to 0.56)	0.474		
Tanner stage 1: reference	-	-	-	-
Tanner stage (II vs. I)	-0.63 (-0.78 to -0.48)	<0.001		
Tanner stage (III vs. I)	-0.63 (-0.82 to -0.44)	<0.001		
Tanner stage(IV vs. I)	-0.9 (-1.29 to -0.51)	<0.001		
Tanner stage (V vs. I)	-1.4 (-2.76 to -0.04)	0.044		

Results show the adjusted differences in z-scores between HIV groups. The model with random intercept only was optimal. Multi-predictor analysis is shown after adjusting for sex, age and height.

CHU: Children who are HIV unexposed; CHEU: Children who are HIV exposed uninfected; CHIV: Children living with perinatally-acquired HIV

Gie A, *et al. Thorax* 2023; 78:1233–1239. doi: 10.1136/thorax-2023-220197

Supplementary Table 8: RV/TLC z-score

Association between each HIV group and RV/TLC z-score using linear mixed effects model. Unadjusted estimates show single-predictor analysis of each variable on RV/TLC z-score. Adjusted results show the mean difference (with 95% CI) in RV/TLC z-score between the exposed group (CHIV/CHEU) and the unexposed group, CHU.

Predictors	Unadjusted Estimate (95% CI)	p-value	Adjusted Estimate (95% CI)	p-value
CHIV (vs CHUU)	0.22 (0.02 to 0.41)	0.034	0.11 (-0.09 to 0.31)	0.296
CHEU (vs CHUU)	0.14 (-0.07 to 0.35)	0.198	0.14 (-0.07 to 0.35)	0.188
Sex: Male	0.03 (-0.13 to 0.18)	0.724	-	-
Age (per year)	-0.22 (-0.24 to -0.19)	<0.001	-0.16 (-0.22 to -0.09)	<0.001
Height (per 1cm increase)	-0.04 (-0.04 to -0.03)	<0.001	-0.01 (-0.02 to -0.00)	0.040
Weight (per 1kg increase)	-0.04 (-0.05 to -0.03)	<0.001	-	-
BMI z-score	-0.07 (-0.13 to 0)	0.059	-0.07 (-0.14 to 0.01)	0.072
Cotinine group: <10ng/mL (reference)	-	-	-	-
11-30ng/mL	0.16 (-0.03 to 0.36)	0.102	0.14 (-0.05 to 0.33)	0.181
30-100 ng/mL	0 (-0.25 to 0.26)	0.970	0.12 (-0.14 to 0.37)	
>100 ng/mL	0.47 (-0.03 to 0.97)	0.068	0.42 (-0.07 to 0.91)	
Tanner stage 1: reference	-	-	-	-
Tanner stage (II vs. I)	-0.74 (-0.92 to -0.56)	<0.001	-	-
Tanner stage (III vs. I)	-0.84 (-1.06 to -0.61)	<0.001	-	-
Tanner stage(IV vs. I)	-1.2 (-1.67 to -0.74)	<0.001	-	-
Tanner stage (V vs. I)	-1.59 (-3.24 to 0.06)	0.059	-	-

Results show the adjusted differences in z-scores between HIV groups. The model with random intercept only was optimal. Multi-predictor analysis is shown after adjusting for height, age, BMI z-score and cotinine group.

CHU: Children who are HIV unexposed; CHEU: Children who are HIV exposed uninfected; CHIV: Children living with perinatally-acquired HIV

Gie A, *et al. Thorax* 2023; 78:1233–1239. doi: 10.1136/thorax-2023-220197

Supplementary Table 9: VC z-score

Association between each HIV group and VC z-score using linear mixed effects model.

Unadjusted estimates show single-predictor analysis of each variable on VC z-score.

Adjusted results show the mean difference (with 95% CI) in VC z-score between the exposed group (CHIV/CHEU) and the unexposed group, CHU.

<table>
<tr><th>Predictors</th><th>Unadjusted Estimate (95% CI)</th><th>p-value</th><th>Adjusted Estimate (95% CI)</th><th>p-value</th></tr>
<tr><td>CHIV (vs CHUU)</td><td>0.09 (-0.17 to 0.34)</td><td>0.493</td><td>0.19 (-0.06 to 0.44)</td><td>0.134</td></tr>
<tr><td>CHEU (vs CHUU)</td><td>-0.15 (-0.41 to 0.11)</td><td>0.244</td><td>-0.11 (-0.37 to 0.15)</td><td>0.406</td></tr>
<tr><td>Sex: Male</td><td>0.21 (0.02 to 0.41)</td><td>0.035</td><td>0.24 (0.05 to 0.44)</td><td>0.015</td></tr>
<tr><td>Age (per year)</td><td>0.09 (0.06 to 0.12)</td><td><0.001</td><td>0.1 (0.06 to 0.13)</td><td><0.001</td></tr>
<tr><td>Height (per 1cm increase)</td><td>0.02 (0.01 to 0.02)</td><td><0.001</td><td></td><td></td></tr>
<tr><td>Weight (per 1kg increase)</td><td>0.02 (0.02 to 0.03)</td><td><0.001</td><td></td><td></td></tr>
<tr><td>BMI z-score</td><td>0.13 (0.05 to 0.21)</td><td>0.001</td><td>0.17 (0.09 to 0.24)</td><td><0.001</td></tr>
<tr><td>Cotinine group: <10ng/mL (reference)</td><td>-</td><td>-</td><td>-</td><td>-</td></tr>
<tr><td>11-30ng/mL</td><td>-0.28 (-0.52 to -0.03)</td><td>0.028</td><td>-0.19 (-0.43 to 0.05)</td><td rowspan="3">0.115</td></tr>
<tr><td>30-100 ng/mL</td><td>-0.05 (-0.36 to 0.27)</td><td>0.764</td><td>-0.03 (-0.34 to 0.29)</td></tr>
<tr><td>>100 ng/mL</td><td>-0.56 (-1.2 to 0.08)</td><td>0.086</td><td>-0.60 (-1.20 to 0.01)</td></tr>
<tr><td>Tanner stage 1: reference</td><td>-</td><td>-</td><td>-</td><td>-</td></tr>
<tr><td>Tanner stage (II vs. I)</td><td>0.17 (0 to 0.34)</td><td>0.045</td><td></td><td></td></tr>
<tr><td>Tanner stage (III vs. I)</td><td>0.4 (0.19 to 0.62)</td><td><0.001</td><td></td><td></td></tr>
<tr><td>Tanner stage(IV vs. I)</td><td>0.51 (0.03 to 0.99)</td><td>0.038</td><td></td><td></td></tr>
<tr><td>Tanner stage (V vs. I)</td><td>0.24 (-1.74 to 2.22)</td><td>0.812</td><td></td><td></td></tr>
</table>

Results show the adjusted differences in z-scores between HIV groups. The model with random intercept and random slope with age was optimal. Multi-predictor analysis is shown after adjusting for sex, age, BMI z-score and cotinine group.

CHU: Children who are HIV unexposed; CHEU: Children who are HIV exposed uninfected; CHIV: Children living with perinatally-acquired HIV

Gie A, *et al. Thorax* 2023; 78:1233–1239. doi: 10.1136/thorax-2023-220197

Supplementary Table 10: D_{LCO} z-score

Association between each HIV group and D_{LCO} z-score using linear mixed effects model.

Unadjusted estimates show single-predictor analysis of each variable on D_{LCO} z-score.

Adjusted results show the mean difference (with 95% CI) in D_{LCO} z-score between the exposed group (CHIV/CHEU) and the unexposed group, CHU.

Predictors	Unadjusted Estimate (95% CI)	p-value	Adjusted Estimate (95% CI)	p-value
CHIV (vs CHUU)	0.00 (-0.31 to 0.3)	0.994	0.03 (-0.24 to 0.31)	0.822
CHEU (vs CHUU)	-0.02 (-0.34 to 0.3)	0.910	0.03 (-0.26 to 0.32)	0.843
Sex: Male	-0.03 (-0.27 to 0.21)	0.827		
Age (per year)	-0.09 (-0.15 to -0.04)	0.001	-0.12 (-0.18 to -0.05)	<0.001
Height (per 1cm increase)	-0.02 (-0.03 to -0.01)	0.001		
Weight (per 1kg increase)	-0.01 (-0.02 to 0)	0.094		
BMI z-score	0.07 (-0.03 to 0.17)	0.183		
Cotinine group: <10ng/mL (reference)	-	-	-	-
11-30ng/mL	0.03 (-0.27 to 0.34)	0.821		
30-100 ng/mL	0.16 (-0.23 to 0.55)	0.413		
>100 ng/mL	-0.07 (-0.99 to 0.85)	0.879		
Tanner stage 1: reference	-	-	-	-
Tanner stage (II vs. I)	-0.47 (-0.78 to -0.16)	0.003		
Tanner stage (III vs. I)	-0.24 (-0.62 to 0.14)	0.207		
Tanner stage(IV vs. I)	-0.33 (-0.95 to 0.29)	0.297		
Tanner stage (V vs. I)	-1.06 (-3.15 to 1.02)	0.316		

Results show the adjusted differences in z-scores between HIV groups. The model with random intercept and random slope with age was optimal. Multi-predictor analysis is shown after adjusting for age.

CHU: Children who are HIV unexposed; CHEU: Children who are HIV exposed uninfected; CHIV: Children living with perinatally-acquired HIV

Gie A, *et al. Thorax* 2023; 78:1233–1239. doi: 10.1136/thorax-2023-220197

Supplementary Table 11: V_A z-score

Association between each HIV group and V_A z-score using linear mixed effects model. Unadjusted estimates show single-predictor analysis of each variable on V_A z-score. Adjusted results show the mean difference (with 95% CI) in V_A z-score between the exposed group (CHIV/CHEU) and the unexposed group, CHU.

Predictors	Unadjusted Estimate (95% CI)	p-value	Adjusted Estimate (95% CI)	p-value
CHIV (vs CHUU)	0.17 (-0.15 to 0.49)	0.301	-0.26 (-0.72 to 0.19)	0.252
CHEU (vs CHUU)	0.11 (-0.23 to 0.44)	0.531	-0.42 (-0.89 to 0.12)	0.080
Sex: Male	0.16 (-0.09 to 0.42)	0.215	-0.35 (-0.84 to 0.13)	0.153
Age (per year)	-0.16 (-0.21 to -0.11)	<0.001		
Height (per 1cm increase)	-0.03 (-0.04 to -0.02)	<0.001	-0.05 (-0.06 to -0.03)	<0.001
Weight (per 1kg increase)	-0.03 (-0.04 to -0.01)	<0.001	0.02 (0.00 to 0.04)	0.020
BMI z-score	0.04 (-0.06 to 0.15)	0.424		
Cotinine group: <10ng/mL (reference)	-	-	-	-
11-30ng/mL	-0.14 (-0.46 to 0.17)	0.371	-0.10 (-0.41 to 0.22)	
30-100 ng/mL	0.23 (-0.18 to 0.64)	0.265	0.38 (-0.01 to 0.78)	0.177
>100 ng/mL	-0.06 (-0.99 to 0.87)	0.894	-0.00 (-0.89 to 0.89)	
Tanner stage 1: reference	-	-	-	-
Tanner stage (II vs. I)	-0.68 (-0.92 to -0.44)	<0.001		
Tanner stage (III vs. I)	-0.63 (-0.96 to -0.3)	<0.001		
Tanner stage(IV vs. I)	-0.98 (-1.55 to -0.4)	0.001		
Tanner stage (V vs. I)	-1.17 (-3.23 to 0.9)	0.266		
Interaction: CHIV*gender (Male)			0.53 (-0.10 to 1.15)	0.026
Interaction: CHEU*gender (Male)			0.87 (0.22 to 1.53)	

Results show the adjusted differences in z-scores between HIV groups. The model with random intercept only was optimal. Multi-predictor analysis is shown after adjusting for gender, height, weight, cotinine group, and the interaction between HIV group and gender. CHU: Children who are HIV unexposed; CHEU: Children who are HIV exposed uninfected; CHIV: Children living with perinatally-acquired HIV
